# Hydroxysafflor Yellow A Ameliorates Renal Fibrosis by Suppressing TGF-β1-Induced Epithelial-to-Mesenchymal Transition

**DOI:** 10.1371/journal.pone.0153409

**Published:** 2016-04-18

**Authors:** Naping Hu, Jialin Duan, Huihui Li, Yanhua Wang, Fang Wang, Jianjie Chu, Jin Sun, Meiyou Liu, Chao Wang, Chengtao Lu, Aidong Wen

**Affiliations:** 1 Department of Pharmacy, Xijing Hospital, Fourth Military Medical University, Xi’an, 710032, China; 2 College of Pharmacy, Shaanxi University of Chinese Medicine, Xianyang, 712046, China; Hunter College of The City University of New York, UNITED STATES

## Abstract

**Objective:**

Renal fibrosis is the common pathological foundation of many chronic kidney diseases (CKDs). The aim of this study was to investigate whether Hydroxysafflor yellow A (HSYA) can preserve renal function by inhibiting the progression of renal fibrosis and the potential mechanisms.

**Methods:**

Renal fibrosis was induced by unilateral ureteral obstruction (UUO) performed on 7-week-old C57BL/6 mice. HSYA (10, 50 and 100 mg/kg) were intragastrically administered. Sham group and model group were administered with the same volume of vehicle. Serum and kidney samples were collected 14 days after the UUO surgery. Serum biochemical indicators were measured by automatic biochemical analyzer. Histological changes were evaluated by HE and Masson staining. *In vitro*, the anti-fibrotic effect of HSYA was tested on human recombinant transforming growth factor-β1 (TGF-β1) stimulated HK-2 cells. The protein levels of α-SMA, collagen-I and fibronectin in kidney tissue andHK-2 cells were measured by immunohistochemistry and immunofluorescence. The protein levels of apoptosis-relative and TGF-β1/Smad3 signaling were detected by western blot.

**Results:**

HSYA slowed the development of renal fibrosis both *in vivo* and *in vitro*. In UUO rats, renal function index suggested that HSYA treatment decreased the level of serum creatinine (Scr) and blood urea nitrogen (BUN) rose by UUO (*P*<0.05). HE staining and Masson staining demonstrated that kidney interstitial fibrosis, tubular atrophy, and inflammatory cell infiltration were notably attenuated in the high-dose HSYA group compared with the model group. The expressions of α-SMA, collagen-I and fibronectin were decreased in the UUO kidney and HK-2 cells of the HSYA-treatment group. Moreover, HSYA reduced the apoptotic rate of HK-2 cells stimulated by TGF-β1. Further study revealed that HSYA regulated the TGF-β1/Smads signaling pathway both in kidney tissue and HK-2 cells.

**Conclusions:**

These results suggested that HSYA had a protective effect against fibrosis in renal cells, at least partly, through inhibiting TGF-β1/smad3-mediated Epithelial–mesenchymal transition signaling pathway.

## Introduction

Chronic kidney disease (CKD) is an increasing public health issue with up to 160 million individuals worldwide predicted to be affected by 2020 [1, 2]. Effective therapeutics is urgently needed to treat this disease and reduce healthcare expenditure. Though the specific mechanism of CKD remains uncertain, renal fibrosis, particularly tubulointerstitial fibrosis is accepted asthe common pathway for chronic kidney disease leading to end-stage renal failure, regardless of etiology[3].Renal fibrogenesis is a dynamic and converging process, characterized by activated tubulointerstitial myofibroblast and the production of excessive extracellular matrix(ECM), and the activated myofibroblasts is believed to be a main contributor in the pathogenesis of renal interstitial fibrosis [4, 5]. Although the exact origins of these myofibroblasts remain uncertain, emerging evidence suggests that they may originated from EMT [6]. During the process of EMT, renal tubular epithelial cells and capillary endothelia transition to a mesenchymal phenotypically and functionally into myofibroblasts[7, 8]. Currently, studies confirmed that EMT regulated by numerous cytokines, and TGF-β1 is considered the major regulator.

TGF-β1, which is widely accepted as an essential fibrogenic cytokine, and its downstream Smad3 has been confirmed an essential role in fibrogenesis[9]. In the injured kidney, it observed that TGF-β1 highly upregulated and its downstream Smad cascade are prevalent with severe renal fibrosis [10]. Evidence shows that TGF-β1 can initiate and complete the entire course of EMT processes, both in patients and animal disease models [11–13]. Furthermore, recent findings indicated that the progress of EMT can be reversible [14]. Hence, intervention of TGF-β1/Smads mediated EMT had been the most intensively targets of various antifibrotic therapies.

HSYA is the main active component of safflower, which has been widely used for the treatment of trauma, cardiovascular and cerebrovascular diseases [15]. Interestingly, in recent years, studies had shown that HSYA might be a promising anti-fibrotic herbal medicine. Experiments suggested that HSYA could attenuate hepatic fibrosis, reduce liver fibrosis and suppress pulmonary fibrosis [16–18]. Further studies also showed thatthe antifibrotic effect was mainly referred to blocking TGF-β1signaling. In addition, HSYA has been used as safflower injection to treat vary kinds of chronic kidney diseases in clinical alone or in conjunction with other medicine in china. These studies suggested that HSYA might be a potential renoprotective agent. Unfortunately, the scientific evidence of HSYA still inadequate and the mechanism is unclear.

Based on above studies, we hypothesized that HSYA might through regulating TGF-β1 signal pathway to ameliorate renal fibrosis in this study. To test the hypothesis, we investigated HSYA in UUO model and TGF-β1–mediated EMT in HK-2 cells. Our findings suggested that HSYA improved renal function by inhibiting the expression of TGF-β1 and Smad3 in injury kidney and TGF-β1–stimulatedHK-2 cell. HSYA has the potential to be developed as a therapeutic agent to prevent renal fibrosis.

## Materials and Methods

### Reagents and Antibodies

HSYA (98%, C_27_H_32_O_16_, m.w. 612.53) was purchased from the Wuben Biological Technology Co (Xi’an, China). Recombinant Human transforming growth factor beta1 (TGF-β1) was purchased from Peprotech (USA); Anti-TGF-β1 antibody was purchased from Cell Signaling Technology (Beverly, MA, USA); Antibodies against smad7, a-SMA, fibronectin, and collagen-I were purchased from the Boster Co (Wuhan, China); Anti-phospho-smad3, anti-smad3,E-cadherin and anti-β-actin antibody were purchased from Biosynthesis Biotechnology Co (Beijing, China); SIS3 was purchased from Santa Cruz Biotechnology (USA).

### Experimental Animals

#### Ethics Statement

All animal experiments were performed in adherence with the National Institutes of Health Guidelines for the Use of Laboratory Animals and were approved by the Fourth Military Medical University Committee on Animal Care. Male C57BL/6 mice (7weeks old, 20–24 g) were obtained from the animal research center at the Fourth Military Medical University, Xi’an, and China (SCXK 2012–0007).

### Establishment of the UUO Model

Prior to the experiments, 30 health male mice were acclimatized for 1 week. They were housed in temperature-controlled conditions, humidity, lighting (12 h light/12 h dark cycle), with free access to food and water. UUO or sham surgery was performed under 5% chloralic hydrasanesthesia, and all effortsin a humane manner as literature described [19]: the right ureter was ligated with 4–0 silk at uretero pelvic junction through a right flank incision, Sham group mice had their ureters exposed and manipulated without ligation, and then the wound was closed in layers. The mice underwent surgery were randomly divided into five groups (six rats per group): sham group, UUO groups, HSYA (10, 50 and 100 mg/kg) treatment group. Mice were given HSYA as the corresponding dose by daily gastric gavage beginning the first day after surgical procedure. Sham group and UUO group were given identical voluminal saline. Both obstructed and contralateral kidneys were harvested 14 days after surgery and either stored at -80°Cor fixed with 4% paraformaldehyde.

### Biochemical Indicators Measurements

The blood samples, obtained from the eyeball after14-day treatment, were centrifuged at 4000 rpm for 10 min then the separated serum was stored in Eppendorf tubes stored at -80°C. Serum creatinine (Scr) and blood urea nitrogen (BUN) levels were measured with7160 automatic biochemical detector (Hitachi Co., Tokyo, Japan).

### HE Staining and Masson Staining

The fixed renal tissues were dehydrated in graded alcohol and then embedded in paraffin. Sections were stained with hematoxylin & eosin (HE) to assess renal injury and Masson staining to assess the level of collagen deposition. Semi-quantitative evaluation was performed at ten non-overlapping vision fields that randomly selected under Olympus microscope (Olympus IX71, Japan)and photographed in each group.

### Immunohistochemistry

Paraffin-embedded sections(5μm) were deparaffinized in xylene and hydrated in graded ethanol, washed with PBS, treated with 3% (v/v) H_2_O_2_ for 15 min, blocked with 10% (w/v) normal goat serum for 1 h, then sections were incubated at 4°C overnight with primary antibodies against a-SMA, and collagen-I, respectively. After washed with PBS, co-incubated with horseradish peroxides (HRP)-labeled goat anti-rabbit/mouse secondary antibody at 37°C for 30 min. DBA colorized for 15min, hematoxylin staining for 3 min, flush 2 min. After conventional dehydration, clearing and mounting with neutral gum, slices were observed under microscope. The Image Pro-Plus Software (Media Cybernetics, Rockville, MD) was employed to calculate the expression of a-SMA and collagen-I.

### Cell Culture and Treatments

In vitro experiments were performed on HK-2 cells, which cultured in DMEM/F12 (1:1) with 10% fetal bovine serum (FBS), 100 U/ml penicillin and 100μg/ml streptomycin in a cell incubator with 5% CO_2_ at 37°C. Fresh growth medium was added to cells every 2 to 3d until confluent.

### Cell Viability Assay

After digested with 0.25% trypsin, 5×10^4^cells were seeded into 96-well plates with complete medium and incubated at 37°Cin 5% CO_2_ overnight. Then HK-2 divided into seven groups:Control group, TGF-β1 (5ng/ml) group, HSYA (1, 10, 50, 100 and 200μg/ml)group. Three time points12h, 24h and 48h were applied to value the viability. MTT (1 mg/ml) solution 100ul was added into each well and then incubated for 4 h in an incubator. Thereafter, the medium was removed from each well before added with 150μl of DMSO, and then shaken for l0 min to dissolve the resulting blue crystals. The absorbance was measured at 570 nm using Microplate Reader (Thermo Fisher Scientific, Shanghai, China). Cell viability was expressed as a percentage of the control (S4 Table).

### Immunocytochemistry

HK-2 cells cultured on coverslips were washed with sterile cold PBS three times and then fixed in 4% paraformaldehyde for 15 min and permeabilized with 0.5% TritonX-100 in PBS for 30 min at room temperature. After fixation, slides were blocked with 1% bovine serum albumin (BSA) for 1 h before washing with PBS. Subsequently slides were incubated with the primary antibody, diluted in 1%BSA overnight at 4°C. After washing, cells were incubated with the appropriate FITC-conjugated secondary and should be lucifugal. Finally, slides were mounted and analyzed by fluorescence microscopy.

### Western Blot Analysis

HK-2 cells were lysed by RIPA lysis buffer containing protease inhibitor PMSF on ice. Then the lysates was collected after centrifugation at 10000 rpm at 4°C for 20 min. Protein concentration were tested with a Coomassie brilliant blue protein quantitative kit (Jiancheng Bioengineering Institute, Nanjing, China) according to the manufacturer’s instructions, and then heated with sample buffer at 100°C for 5 min before loading and separated by sodium dodecyl sulfate–polyacrylamide gel electrophoresis (SDS–PAGE) on 12% polyacrylamide gels. The proteins were electro transferred to a nitro-cellulose (NC) membrane in transfer buffer for 30min. In order to prevent nonspecific background binding, the membraneswere incubated with 5% non-fat milk in Tris-buffered saline with 0.1% Tween 20 (TBST) at room temperature for 2 h. The immunoblots incubated with various primary antibodies in blocking buffer (containing 5% bovine serum albumin, 0.1% Tween 20 in 0.1 TBS, pH 7.4) at the dilutions specified by the manufacturers overnight at 4°C, following three washes with TBST, the membranes were incubated with horseradish peroxidase-conjugated secondary anti-body for 1 h at 37°C. And then an ECL detection kit was used to detect the bound antibodies. Results were normalized to β-actin and quantified with image analysis systems (Bio-Rad, USA).

### Statistical Analysis

All experiments were repeated as mean ± standard deviation (SD). Multiple comparisons were examined for significant differences using ANOVA, followed by individual comparison with the Tukey’ s posthoctest, with *P*<0.05 indicating statistical significant difference.

## Results

### HSYA Decreases the Level of Scr and BUN

**Fig 1:**
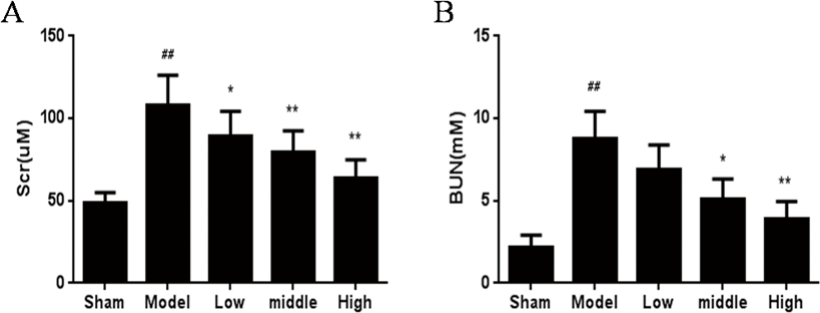
Effect of HSYA on the levels of Scr and BUN in UUO rats. The levels of Scr (A) and BUN (B) were measured by automatic biochemical detector. Sham represent sham group; Model represent UUO group; Low, Middle and High represent UUO rats were treatment with 10, 50 and 100 mg/kg HSYA, respectively, and the high–dose group exhibit an clear effect. Data are expressed as mean±SD, n = 6. ^##^*P*<0.01 compared with sham group; *P<0.05, **P<0.01compared with model group

Scr and BUN are the classical indicators of renal function. The data (S1 Table) showed that Scr and BUN were markedly elevated in the UUO group compared with the sham group and reduced by HSYA. Indicating that UUO model had been successfully established and HSYA exerted a protective effect on renal injury and dysfunction (Fig 1).

### HSYA Alleviates Kidney Tissue Injury in UUO rats

**Fig 2:**
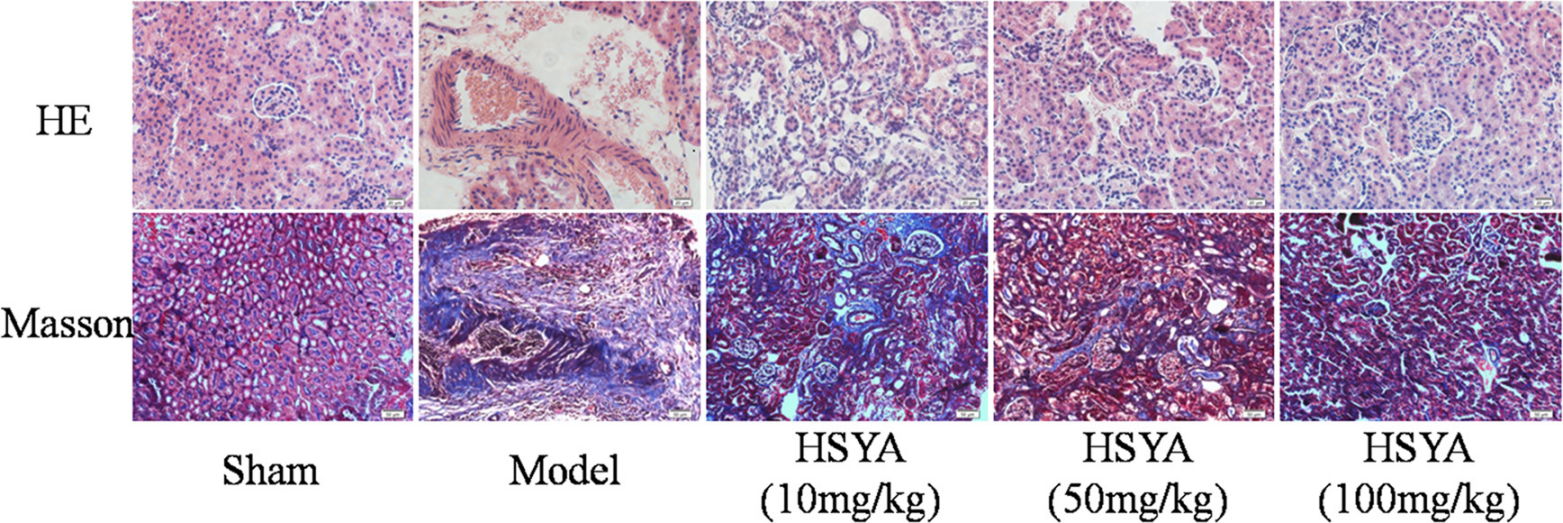
Effect of HSYA on renal histopathology and tubulointerstitial fibrosis induced by UUO. HE staining (magnification×400) showed the ensemble of renal structural damage at 14 days after UUO surgery. Masson staining (magnification×400) illustrated the attenuation of tubulointerstitial fibrosis of kidney tissue after treated by HSYA with different concentrations.

From HE staining, we observed the morphological changes in the UUO model, including tubular atrophy, inflammatory cell infiltration, segmental thickening of glomerular basement membranes, excessively deposited mesangial matrix, and renal interstitial fibrosis remarkable. However, treatment with HSYA attenuated these ultrastructural abnormalities in a dose-dependent manner. Masson staining showed that collagen deposition and fibrosis area were significantly decreased in HSYA treatment groups than in UUO group (Fig 2).

### HSYA Inhibits Kidney Tissue Fibrosis

**Fig 3:**
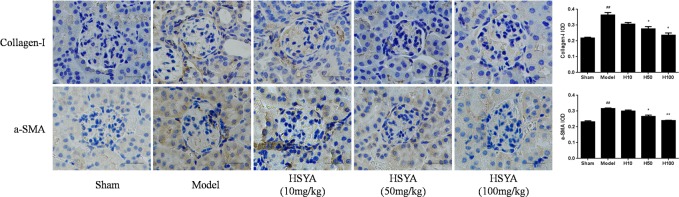
Effect of HSYA on the expression of a-SMA and collagen- I in kidney tissue. Compare with model group, HSYA prevented the increased level of a-SMA and collagen-I in a dose-dependent manner, and the highest concentration significantly suppressed the expressions of a-SMA and collagen-I. Data are expressed as mean±SD, n = 3. ^##^*P*<0.01 compared with sham group, *P<0.05, **P<0.01 compared with model group.

It has been acknowledged that a-SMA and collagen-I are hallmarks of myofibroblasts and excessive extracellular matrix accumulation[20]. Immunohistochemistry and semi-quantitative data (S2 Table) demonstrated that a-SMA and collagen-I were increased in the UUO group and partly reversed after treatment with HSYA. Furthermore, the changes were more obvious in the high-dose group (Fig 3).

### HSYA Prevents TGF-β1-induced Cell Proliferation

**Fig 4:**
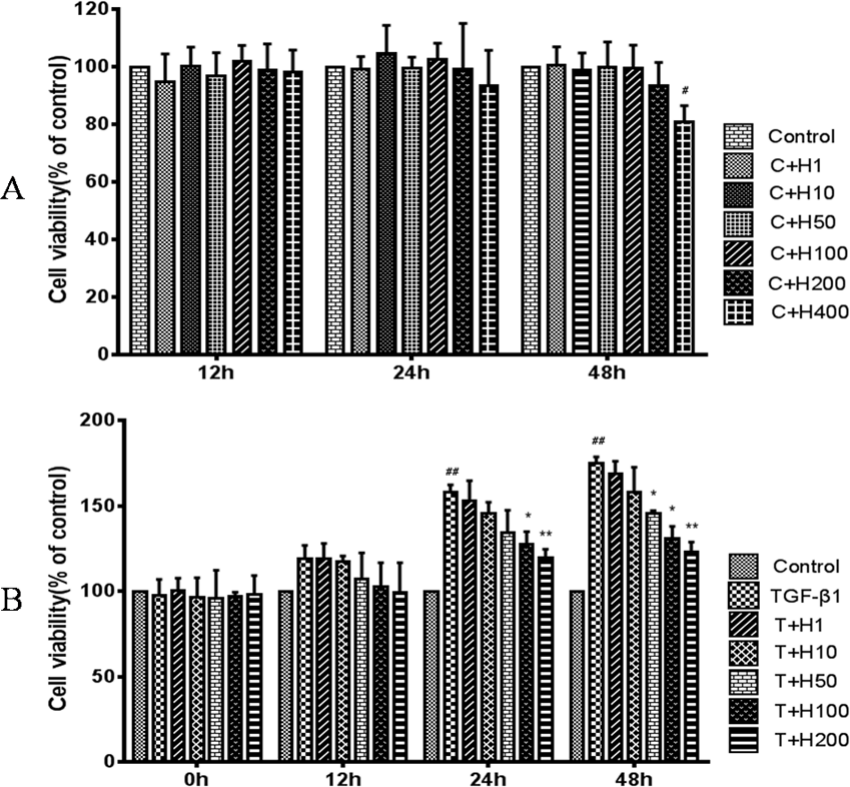
Effect of HSYA on cell proliferation. Cells were incubated for 12h, 24h and 48h in different concentration of HSYA to measure the toxicity (A). The HSYA-treated group stimulated with both recombinant TGF-β1 (5ng/ml) and the different concentrations for three time points (B). Data are expressed as percentage of control group ±SD (n = 3), ^##^*P*<0.01compared with control group; *P<0.05 and **P<0.01compared with TGF-β1-treated group.

To investigate whether HSYA inhibits TGF-β1-induced EMT in renal tubule epithelial cells, we firstly observed the toxicity of HSYA at different concentrations (Fig 4A).Then HK-2 cells were stimulated by human recombinant TGF-β1 (5ng/ml), and treated with different concentrations of HSYA. Cell proliferation was significantly inhibited by HSYA in a dose-dependent manner, and the phenomenon was more prominent at 200μg/ml after 24 hours. Then 200μg/ml was applied in the following experiment (Fig 4B).

### HSYA Attenuates TGF-β1-mediated EMT and ECM Accumulation in HK2 Cells

**Fig 5:**
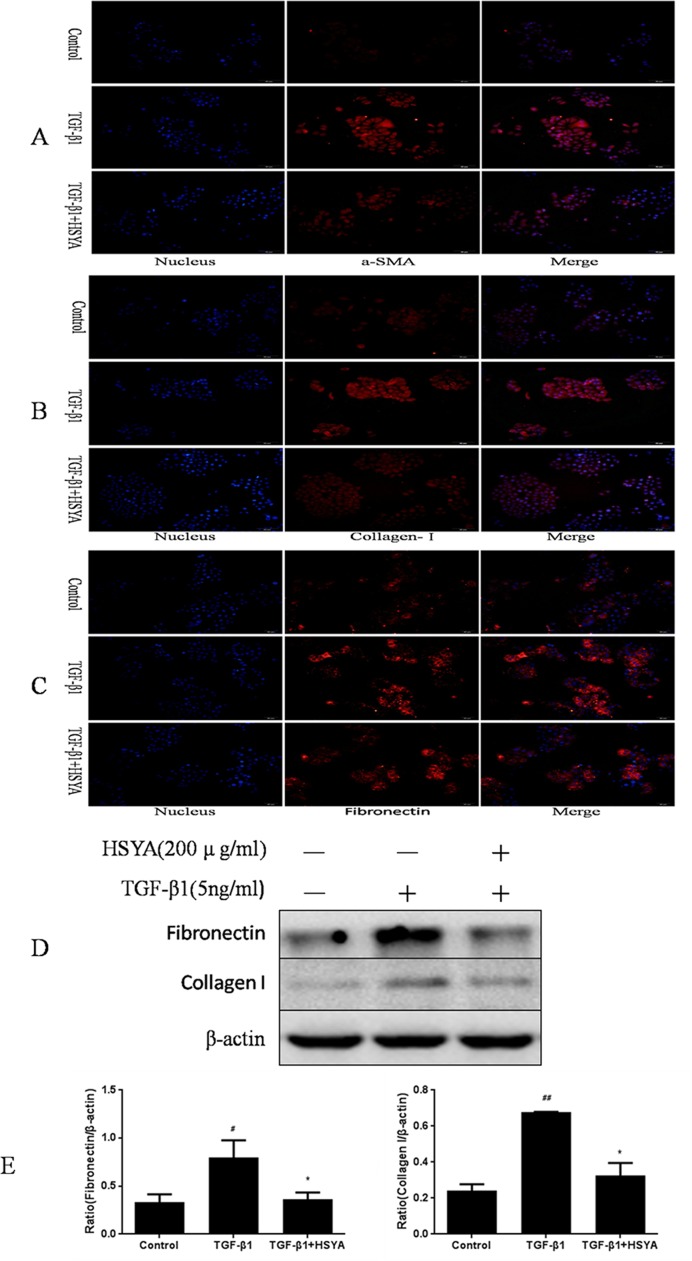
Effect of HSYA on TGF-β1-induced myofibroblasts and accumulation of extracellular matrix in HK-2 cells. Immunofluorescence staining measured the expression levels of a-SMA (A), collagen-I (B) and fibronectin (C). Western blot analysis of FN and collagen-I protein levels (D). Semi-quantitative data from densitometric analysis of FN and collagen-I are presented as relative ratio of each protein to β-actin (E). Data are expressed as mean±SD, n = 3. ^#^*P*<0.05, ^##^*P*<0.01compared with control group; *P<0.05 compared with TGF-β1-stimulated group.

Due to the fact that the progression of EMT paves the way for extracellular matrix (ECM) deposition and finally deteriorate renal fibrosis, we detected the expression of a-SMA, collagen-I and fibronectin by Immunofluorescence. Results showed that the level of a-SMA, collagen-I and fibronectin were notable with TGF-β1 stimulation. However, HSYA treatment obviously down regulated a-SMA and collagen-I protein expression, and slight down regulation of fibronectin (Fig 5A–5C).We further evaluated the expression of fibronectin and collagen-I via Western blot. Results showed that both fibronectin and collagen-I protein were higher in TGF-β1-stimulated group than in control group. HSYA treatment significantly ameliorated TGF-β1-induced increase of fibronectin and collagen-I expression (Fig 5D and 5E). These results indicated that HSYA could prevent the expression of the fibroblast marker and ECM in HK-2 cells, thus slow down the progression of TGF-β1-induced EMT.

### HSAY Suppresses TGF-β1-induced Apoptosis in HK-2 cells

**Fig 6:**
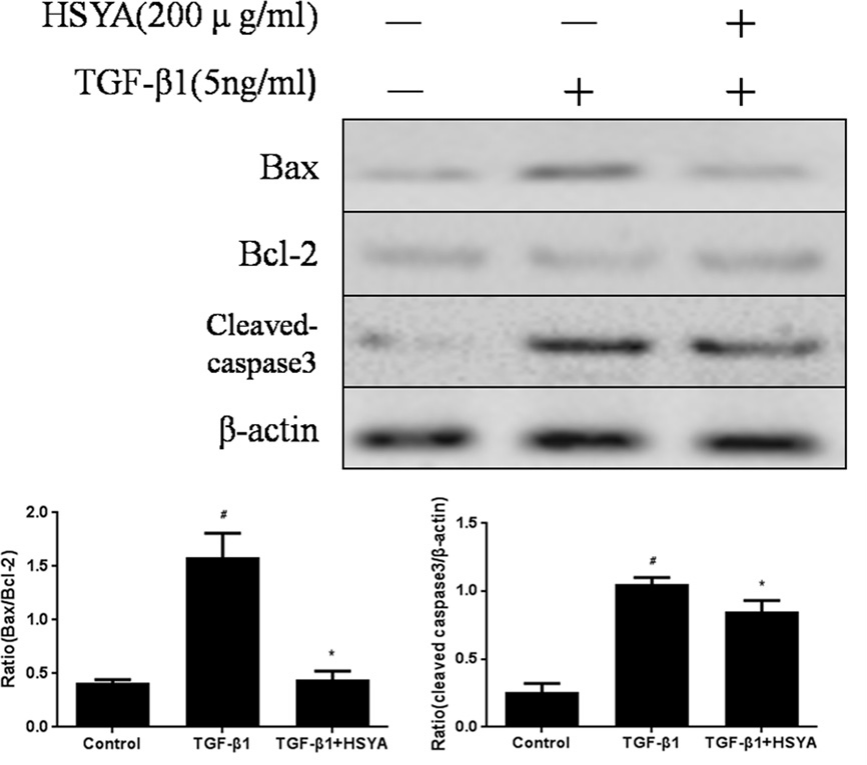
Effect of HSYA on TGF-β1-induced Apoptosis in HK-2 Cells. The apoptosis-relative protein level of Bax, Bcl-2 and cleaved caspase3 were measured. Semi-quantitative data from densitometric analysis of Bax, Bcl-2 and caspase3 are presented as relative ratio of each protein to β-actin. Data are expressed as mean±SD, n = 3, ^#^*P*<0.05 compared with control group; *P<0.05 compared with TGF-β1-stimulated group.

It is well-known that cell apoptosis involved in the injury and repair progress of multiple kidney disease. Growing evidence showed that TGF-β1/Smads signaling could mediate cell apoptosis by stimulating expression of pro-apoptotic members and by suppressing expression of anti-apoptotic members, respectively [21]. Here, we test the expression of pro-apoptotic Bax, anti-apoptotic Bcl-2, and cleaved caspase3 by Western blotting in HK-2 cells. Data (S5 Table) indicated that Bax and caspase3 protein exhibited higher expression in TGF-β1-stimulated group. While, Bcl-2 expression was lower in TGF-β1-stimulated group than in control group. The expression of Bax, Bcl-2 and caspase3 were all reversed in HSYA group. These phenomena suggested that HSYA could reduce cell apoptosis via regulating the ratio of Bax and Bcl-2, down-regulating the expression of cleaved caspase3. (Fig 6)

### HSYA Rebalances the TGF-β1/Smads Signaling in Kidney Tissue and HK-2 Cells

**Fig 7:**
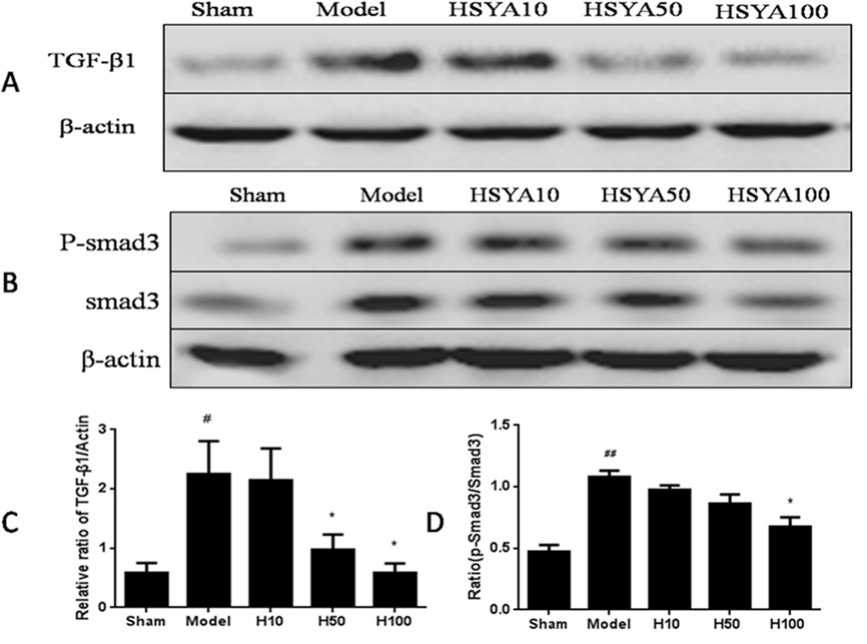
Effect of HSYA on TGF-β1/Smads signaling pathway in kidney tissue. Western blot analysis of TGF-β1 (A), smad3 and phosphorylated Smad3 levels (B). Semi-quantitative data from densitometric analysis of TGF-β1 are presented as relative ratio of each protein to β-actin (C). Semi-quantitative data from densitometric analysis of phospho-Smad3 are presented as the ratio to Smad3 (D). Data are expressed as mean±SD, n = 3, ^#^*P*<0.05, ^##^*P*<0.01compared with Sham group;*P<0.05 compared with model group.

**Fig 8:**
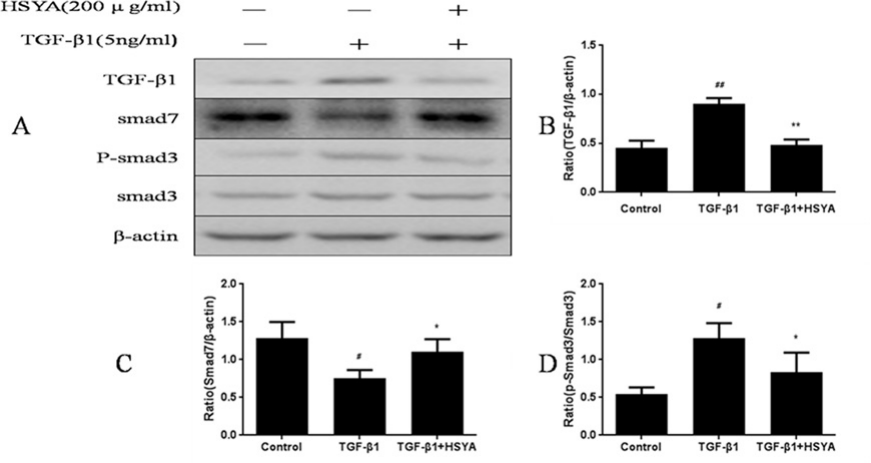
Effect of HSYA on TGF-β1/Smads signaling pathway in HK-2 cells. Western blotting was performed to detect TGF-β1, Smad3, p-Smad3, Smad7 protein levels, and β-actin was used as a loading control (A). Semi-quantitative data from densitometric analysis of TGF-β1 and Smad7 are presented as relative ratio of each protein to β-actin, p-Smad3 are presented as the ratio to Smad3 (B-D). Data are expressed as mean±SD, n = 3, ^#^*P*<0.05,^##^*P*<0.01compared with control group; *P<0.05 compared with TGF-β1-stimulated group.

We then test the effect of HSYA on TGF-β1/Smad3 signaling, which have been confirmed a central role to regulate interstitial fibrosis [22, 23].The level of TGF-β1 was down-regulated compare with the model group, and the ratio of p-Smad3 to Smad3 was decreased in the HSYA-treated groups relative to the model group in a dose-dependent manner (Fig 7 and S3 Table). The semblable effect was identified in HK-2cells. Administration with HSYA notably reduced the high expression of TGF-β1 and p-Smad3 stimulated by TGF-β1. In addition, the negative regulator, Smad7 was up-regulated by HSYA (Fig 8). These results suggested that activation of the TGF-β1/Smads signaling pathway plays a prominent role in the progress of renal fibrosis and it was meaningful that HSYA could rebalance the TGF-β1/Smad ssignaling to exhibiting antifibrotic effect.

### Activation of the TGF-β1/smad3 Pathway Plays a Prominent Role in the Antifibrotic Effect of HSYA

**Fig 9:**
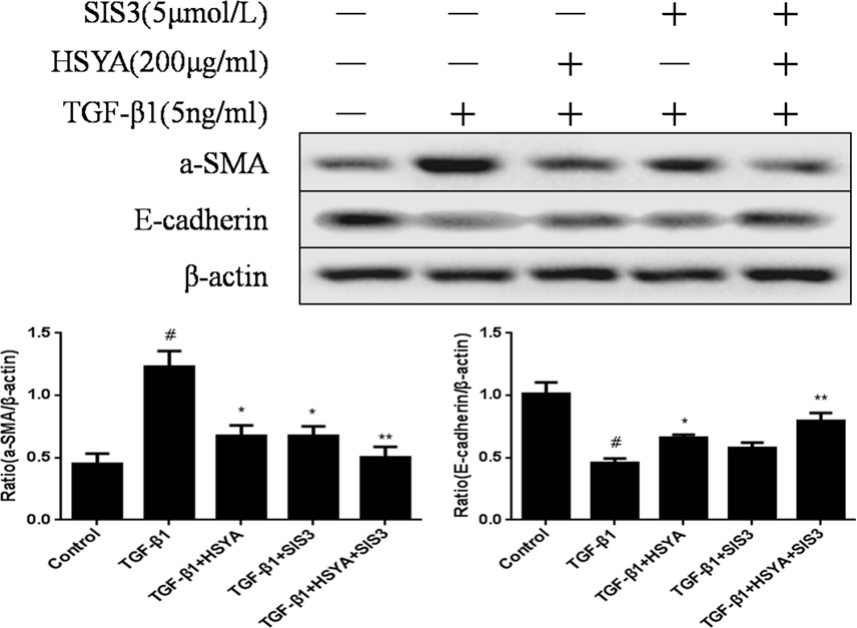
Effect of HSYA and SIS3 onthe expression of α-SMA and E-cadherin in HK-2 cells. HK-2 cells treated with HSYA and SIS3for 48 h after stimulated with TGF-β1 (5 ng/ml). The a-SMA and E-cadherin expression level were detected by western blot. β-actin was used as a loading control. Semi-quantitative data from densitometric analysis of a-SMA and E-cadherin are presented as mean±SD, n = 3. ^#^*P*<0.05 compared with control group; *P<0.05,**P<0.01 compared with TGF-β1-stimulated group.

E-cadherin (the epithelial cell marker) and α-SMA (a specific myofibroblast marker) are usual biomarker of EMT. To further demonstrate the role of HSYA in the EMT process through TGF-β1/smads pathway, we treated HK-2withHSYA and SIS3 (5μM) after stimulated by human recombinant TGF-β1 (5ng/ml)and then to measure the expression of a-SMA and E-cadherin (Fig 9). The results showed that the expression level of a-SMA was reduced and level of E-cadherin was increased in treatment group of HSYA and SIS3, indicating that TGF-β1–induced EMT was reversed by inhibiting the smad3 signaling pathway (S6 Table). This suggested that activation of the TGF-β1/smad3 pathway appears to play a prominent role in the antifibrotic effect of HSYA.

## Discussion

Chronic kidney disease (CKD) is a major healthcare burden and a dominant cause of death worldwide. According to the latest national survey, chronic kidney disease (CKD) has become an important health problem in china [24].Current treatment options primarily targeted at the renin angiotensin system in modulating renal progression modestly, and the urgent need for additional effective therapeutics is evident [25, 26].In recent years, accumulating evidence suggested that renal fibrosis is the major pathological feature of progressive kidney disease and plants used in tradition Chinese medicine and compounds isolated from medicinal plants possess potent anti-fibrotic properties [27–31].In present study, we demonstrated HSYA, the monomer component extracted from safflower has the anti-fibrotic effect, and the mechanism was involved in suppressing TGF-β1-mediated EMT.

It was widely accepted that no matter in clinical patients or in animal studies, renal interstitial fibrosis is the primary pathological feature of kinds of kidney disease, which involves multiple mechanisms. Among the mechanisms, EMT has been recognized as a typical event and gained high attention [32–34]. Renal tubular EMT is a process during which tubular epithelial cells could differentiate into myofibroblasts. This phenotypic conversion not only characterized by activation of a-SMA but also fundamentally leads to excessive synthesis and secretion of extracellular matrix (ECM) proteins, thus thickening and stiffening the basement membrane [35, 36]. In the current study, we found that HSYA could suppress the expression of a-SMA and ECM in kidney tissue and TGF-β1-stimulated HK-2 cells.

TGF-β1was a key pro-fibrotic mediator and mainly activated a ubiquitous intracellular signaling cascade of smads family protein to exert its biological activities such as cell growth, differentiation, ECM production, and apoptosis in renal disease [21, 37]. During renal fibrogenesis, the active TGF-β1 phosphorylates the downstream receptor-associated Smad3to induce EMT and collagen accumulation [38, 39].Moreover, Smad3 associates with an inhibitory Smad, Smad7, which can exert its negative effect on TGF-β1/Smad signaling. Accumulated evidences showed that rebalancing the TGF-β1/Smad signaling by down-regulating Smad3 activity, up-regulating Smad7 may be an effective therapy with fewer side effects [40–45].To test the effect of HSYA on the above-mentioned mechanism, *In vivo*, the representative animal model-UUO model was performed in our study. Results showed that HSYA improved renal function by reducing the raised level of Scr and BUN, and ameliorated UUO-induced pathological change in a concentration-dependent manner. *In vitro*, the protective effect of HSYA was observed in HK-2 cells. Data confirmed that HSYA rebalanced the expression of Smad3 and Smad7in TGF-β1-mediated HK-2 cells. In addition, SIS3, a specific inhibitor of smad3, was used. The results revealed that HSYA, as well as SIS3, significantly reversed EMT induced by TGF-β1 in HK-2 cells, as evidenced by the restoration of diminished E-cadherin and increased α-SMA.

These fibrosis-antagonizing effects were consistent with Yu-Lin Yang, who demonstrated that the crude safflower extract can suppress renal fibrosis by inhibiting the TGF-β autocrine loop[46]. Instead of crude safflower extract, we evaluated the anti-fibrotic effect of HSYA in UUO rats and TGF-β1-stimulate HK-2 cell. Besides, we demonstrated the anti-apoptotic effect of HSAY in HK-2 cells. Results suggested that HSYA may exert its beneficial effects by suppressing cell proliferation and apoptosis in HK-2 cells induced by TGF-β1.Theseverifiedthe report that TGF-β1 mediates not only fibrosis, but also apoptosis, and the anti-apoptotic effect of HSAY may be another effective target for slow down the renal fibrosis process [47].HSYA, as the monomer component extracted from safflower, has been identified the major medicinal substance in the hydrophilic fraction [48].Our findings demonstrate that HSYA is the primary monomer of safflower that plays an important role in suppressing renal fibrosis.

In summary, our findings provide the first evidence that HSYA can be developed as a novel therapeutic agent to delaying the progression of renal fibrosis through inhibition of the TGF-β1/Smads pathway. To clarify the exact mechanism of HSYA inhibiting kidney fibrosis, other underlying molecular mechanisms would be explored in further investigation. Undoubtedly, elucidating the relative contribution of each individual pathway of HSYA to its overall renal protection remains a challenging and meaningful event.

## Supporting Information

S1 Table The levels of Scr and BUN in UUO rats. (XLSX)

S2 Table Semi-quantitative data of collagen-I and a-SMA in UUO rats. (XLSX)

S3 Table Semi-quantitative data of TGF-β1 and p-smad3/smad3 in UUO rats. (XLSX)

S4 Table The effect of HSYA on cell viability. (XLSX)

S5 Table Semi-quantitative data of fibronectin, collagen-I, bax/bcl-2, cleaved caspase-3,TGF-β1, p-smad3/smad3 and smad7 in HK-2 cells. (XLSX)

S6 Table Semi-quantitative data of a-SMA and E-cadherin in HK-2 cells. (XLSX)

## Abbreviations

HSYA: Hydroxysafflor yellow A
CKD: chronic kidney disease
UUO: unilateral ureteral obstruction
TGF-β1: transforming growth factor-β1
EMT: epithelial-to-mesenchymal transition
Scr: Serum creatinine
BUN: blood urea nitrogen
p-smad3: phosphorylated-smad3
a-SMA: a-smooth muscle actin
HE: hematoxylin & eosin
MTT: 3-[4,5-dimethylthiazol-2-yl]-2,5-diphenyltetrazolium bromide
SDS–PAGE: sodium dodecyl sulfate–polyacrylamide gel electrophoresis
PBS: phosphate-buffered saline
DMSO: dimethyl sulfoxide
